# Plasticity of γδ T Cells: Impact on the Anti-Tumor Response

**DOI:** 10.3389/fimmu.2014.00622

**Published:** 2014-12-08

**Authors:** Virginie Lafont, Françoise Sanchez, Emilie Laprevotte, Henri-Alexandre Michaud, Laurent Gros, Jean-François Eliaou, Nathalie Bonnefoy

**Affiliations:** ^1^U896, Institut de Recherche en Cancérologie de Montpellier (IRCM), INSERM, Montpellier, France; ^2^Centre Régional de Lutte Contre le Cancer CRLC Val d’Aurelle – Paul Lamarque, Université Montpellier 1, Montpellier, France; ^3^Département d’Immunologie, Centre Hospitalier Régional Universitaire de Montpellier et Faculté de Médecine, Université Montpellier 1, Montpellier, France

**Keywords:** plasticity, γδ T cells, cytokines, anti-tumor response, pro-tumor response

## Abstract

The tumor immune microenvironment contributes to tumor initiation, progression, and response to therapy. Among the immune cell subsets that play a role in the tumor microenvironment, innate*-like* T cells that express T cell receptors composed of γ and δ chains (γδ T cells) are of particular interest. γδ T cells can contribute to the immune response against many tumor types (lymphoma, myeloma, melanoma, breast, colon, lung, ovary, and prostate cancer) directly through their cytotoxic activity and indirectly by stimulating or regulating the biological functions of other cell types required for the initiation and establishment of the anti-tumor immune response, such as dendritic cells and cytotoxic CD8+ T cells. However, the notion that tumor-infiltrating γδ T cells are a good prognostic marker in cancer was recently challenged by studies showing that the presence of these cells in the tumor microenvironment was associated with poor prognosis in both breast and colon cancer. These findings suggest that γδ T cells may also display pro-tumor activities. Indeed, breast tumor-infiltrating γδ T cells could exert an immunosuppressive activity by negatively regulating dendritic cell maturation. Furthermore, recent studies demonstrated that signals from the microenvironment, particularly cytokines, can confer some plasticity to γδ T cells and promote their differentiation into γδ T cells with regulatory functions. This review focuses on the current knowledge on the functional plasticity of γδ T cells and its effect on their anti-tumor activities. It also discusses the putative mechanisms underlying γδ T cell expansion, differentiation, and recruitment in the tumor microenvironment.

## Introduction

Cancer initiation, progression, and invasion rely on the active communication between cancer cells and the different cell types in the tumor microenvironment, such as fibroblasts, endothelial cells, and immune cells. It is now well established that the immune contexture of the tumor microenvironment can influence cancer progression and outcome (1). All subsets of immune cells can be found within tumors, but their density, functionality, and organization vary according to the tumor type and stage and also from patient to patient. Within the tumor microenvironment, several sub-populations of effector cells participate in controlling and eliminating cancer cells. Among them, innate*-like* T cells that express T cell receptors (TCR) composed of γ and δ chains actively contribute to the anti-tumor immune response in many tumors (lymphoma, myeloma, melanoma, breast, colon, lung, ovary, and prostate cancer) (2–12). They can do this directly through their cytotoxic activity against tumor cells, or indirectly by stimulating and regulating the biological functions of other immune cell types, such as dendritic cells (DC) or interferon γ (IFN-γ)-producing CD8+ T cells, required for the initiation and establishment of an efficient anti-tumor immune response.

γδ T cells belong to the non-conventional or innate lymphocyte family. They differ from conventional αβ T cells, since most of γδ T cells do not express the CD4 and CD8 co-receptors and, as a consequence, antigen recognition by γδ TCR is not restricted to major histo-compatibility complex (MHC) molecules (13, 14). Thus, while αβ TCR interact with peptides bound to MHC class I or class II molecules, γδ TCR recognize a diverse array of self and non-self antigens, such as small peptides, soluble or membrane proteins, phospholipids, prenyl pyrophosphates, and sulfatides. Because of this antigenic diversity, a single mechanism might not explain all observed TCR-dependent γδ T cell responses (15). Moreover as γδ T cell activation does not require antigen processing and presentation by antigen-presenting cells (APC), γδ T cells can be rapidly activated and act during the early phase of the immune response. Like natural killer (NK) cells, γδ T cells also respond to stimulation by stress- and/or infection-induced ligands, such as the MHC class I-related molecules H60, RAE1, and MULT-1 in mice (16), or MICA/B and ULBP in humans (17). Normally, these ligands are weakly or not expressed, they are up-regulated only in the presence of stress (DNA damage, heat stress) or infection and activate γδ T cells by binding to the activating NKG2D receptor expressed on these cells (18–21) and, in some cases, through direct recognition by human γδ TCR (22, 23). Moreover, human γδ T cells also express pattern recognition receptors (PRR), such as Toll-like receptors (TLR), which modulate their activation (24).

In humans, γδ T cells represent 0.5–16% (on average: 4%) of all CD3+ cells in adult peripheral blood, in organized lymphoid tissues (thymus, tonsil, lymph nodes, and spleen), <5% in tongue and reproductive tract and 10–30% in intestine (25, 26). In adult mice, 1–4% of all T cells in thymus, secondary lymphoid organs and lung are γδ T cells. γδ T cells are more abundant in other mucosal sites. Indeed, they constitute 10–20% of all T cells in female reproductive organs (27), 20–40% of the intestinal intraepithelial T cells (28) and 50–70% of skin dermal T cells (29, 30). Moreover γδ TCR repertoire is restricted and depends on the tissue type and their localization. Specifically, Vγ9Vδ2 TCR are expressed by 50–95% of γδ T cells from human peripheral blood (31), whereas, TCR including other Vδ elements are predominantly found in intestinal (Vδ1 and Vδ3) or skin (Vδ1) γδ T cells (32, 33). In mice, γδ T cells with distinct Vγ/Vδ usage are present in spleen (Vγ1 and Vγ4), skin and intestine (Vγ7Vδ4, Vγ7Vδ5, and Vγ7Vδ6), lung (Vγ4 and Vγ6), and reproductive organs (Vγ6Vδ1) (33, 34). While both αβ and γδ T cell subsets are found in human skin (35), γδ T cells expressing the invariant Vγ5Vδ1 are the major population found in mice skin. They form a dense network of dendritic-like cells that are called dendritic epidermal T cells (DETCs) (36).

γδ T cells share many functional characteristics with conventional effector αβ T cells, for instance human Vγ9Vδ2 T cells can display cytotoxic activity against infected or transformed cells and produce pro-inflammatory cytokines, such as tumor necrosis factor α (TNF-α), IL-17, and IFN-γ (33, 34, 37). A unique feature of human Vγ9Vδ2 T cells is the TCR-dependent recognition of non-peptidic phosphorylated antigens, called phosphoantigens. Natural phosphoantigens, such as (*E*)-4-hydroxy-3-methyl-but-2-enyl pyrophosphate (HMBPP) are produced by many bacteria through the prokaryotic isoprenoid pathway (also called non-mevalonate isoprenoid pathway or Rohmer pathway) and are extremely potent activators of human Vγ9Vδ2 T cells (38, 39). On the other hand, eukaryotic cells use the mevalonate isoprenoid pathway to produce phosphoantigens, such as isopentenyl pyrophosphate (40), which are much less active than the natural phosphoantigens produced by bacteria. As the mevalonate pathway plays a key role in multiple cellular processes, the increased metabolism of tumor cells stimulates the over-production and secretion of endogenous phosphoantigens that are sensed by human γδ T cells as tumor-related antigens (40). Through their unique capacity to recognize phosphoantigens, Vγ9Vδ2 T cells play an essential role in anti-infection immunity and also in tumor immune surveillance (41, 42).

Vγ9Vδ2 T cells have rapidly emerged as an attractive therapeutic target for anti-tumor therapies. Indeed, they display a very efficient, non-MHC restricted lytic activity against a broad panel of tumors, they abundantly produce IFN-γ and can be easily expanded from peripheral blood with agonist molecules. Many clinical trials have been carried out based on the adoptive transfer of *in vitro* stimulated Vγ9Vδ2 T cells or on the *in vivo* stimulation of their activity using clinical-grade agonists (43, 44). So far, no concluding result has been obtained from clinical trials based on the adoptive transfer of expanded autologous Vγ9Vδ2T cells; however, *in vivo* stimulation of γδ T cells showed objective responses in 10–33% of patients (45). Although, the lack of response to therapy could be attributed, in some cases, to deficient expansion of effector Vγ9Vδ2 T cells (5, 10, 12), many patients who did not respond to the treatment exhibited significant and sustained Vγ9Vδ2 T cell activation and proliferation. These results suggest that the current γδ T cell-based treatments are feasible and safe, but have some obvious limitations. Thus, a better understanding of effector γδ T cell regulation is required to improve their efficacy (45). Interestingly, recent *in vitro* and *in vivo* data highlighted that γδ T cells show some degree of plasticity driven by environmental signals that can affect and modify their anti-tumor functions and limit their efficacy. Therefore, much research effort is currently focused on precisely understanding the molecular mechanisms that govern the functional plasticity of Vγ9Vδ2 T cells and other γδ T sub-populations and the role of cancer cells and of the tumor microenvironment on the recruitment, polarization, and biological functions of such cells. This knowledge is required to develop optimal strategies for the expansion of γδ T cells with anti- rather than pro-tumor activity.

Here, we provide an overview of the current knowledge on γδ T cell functional plasticity and its effect on their tumor activities. We also discuss the putative mechanisms that underlie γδ T cell expansion, differentiation, and recruitment in the tumor microenvironment.

## Functional Plasticity of γδ Cells

The differentiation of conventional αβ T cells into effector cells is driven by TCR engagement and specific environmental signals. For example, naive αβ CD4 T cells can differentiate into Th1 or Th2 cells following priming by viruses or extracellular parasites, respectively (46–49). This polarization is stably imprinted by lineage-specific transcription factors to allow the generation of memory T cells with appropriate functions to rapidly eliminate the infectious agents after new exposure. However, recent studies demonstrated considerable flexibility, or plasticity, in T cell fate, unraveling the complex relationships among effector and regulatory αβ T cell sub-populations. Similarly, γδ T cells also present some plasticity that contributes to their functional specialization.

### Plasticity of human Vγ9Vδ2 T cells

Several studies showed that after phosphoantigen activation, peripheral human Vγ9Vδ2 T cells promote a Th1 immune response (50–52) characterized by potent TNF-α and IFN-γ production and cytotoxic responses (53, 54). This Th1 cell-like polarization of Vγ9Vδ2 T cells is probably acquired during their postnatal peripheral expansion upon exposure to environmental microbial antigens. Gibbons and collaborators reported that neonatal γδ T cells can produce IFN-γ and that they acquire the ability to produce TNF-α after 1 month of post-partum environmental exposure (55). However *in vitro*, depending on the cytokines and the γδ TCR stimulus provided, adult Vγ9Vδ2 T cells can be polarized into cells with features associated with Th2 cells, Th17 cells, follicular T helper cells (Tfh), or regulatory T cells (Treg) (56–60) (see Table 1).

**Table 1:** **γδ T cell functional plasticity**.

γδ T cell subsets	TCR activation	Cytokines	Polarization *Transcription factors*	Effector molecules	Reference
Adult blood Vγ9Vδ2 T cells	+	IL-12 or IL-18	Th1-like *T-bet, eomesodermin*	IFN-γ, TNF-α	(56)
	+	IL-4	Th2-like *GATA-3*	IL-4	(56)
	+	IL-15 + TGF-β	Treg-like *Foxp3*	IL-10, TGF-β	(60)
	+	IL-6 + IL-23 + IL-1β + TGF-β + Ahr^a^ agonists	Th17-like *ROR*γ*t*	IL-17	(61)
	+	IL-23 + IL-1β + TGF-β	Th17-like, *ROR*γ*t* Th1/17 like, *ROR*γ*t, T-bet* Th22, *FOX04*	IL-17 IFN-γ, IL-17 IL-22	(62)
	+	IL-2	APC functions *ND*	MHC I and II	(63, 64)
Adult blood and tonsillar Vγ9Vδ2 T cells	+	IL-21	Tfh-like *Bcl6*	IL-4, IL-10, CXCL13	(58, 59)
Th1 Vγ9Vδ2 T cells	−	IFN type I	Th1-like *ND*	IFN-γ	(65)
Cord blood Vγ9Vδ2 T cells	+	IL-6 + IL-1β + TGF-β	Th17-like, *ROR*γ*t* Th22-like, *FOX04*	IL-17 IL-22	(62)
	+	IL-6 + IL-1β+ TGF-β + IL-23	Th1/17 like *ROR*γ*t, T-bet*	IFN-γ, IL-17	(62)
Human Vγ1+ and Vγ2+ thymocytes	−	IL-2 or IL-15	Th1 like *T-bet, eomesodermin*	IFN-γ, TNF-α	(66)
Murine γδ T cells	−	IL-23 + IL-1β	Th17 *ROR*γ*t*	IL-17, IL-21, IL-22	(67)

*^a^Aryl hydrocarbon receptor*.

It has been first demonstrated that, Vγ9Vδ2 T cells can be polarized toward IFN-γ-secreting Th1-like γδ T cells upon activation by IPP in the presence of IL-12 and an anti-IL-4 antibody, or toward IL-4-producing Th2-like γδ T cells upon stimulation by IPP in the presence of IL-4 and an anti-IL-12 antibody (56).

**Figure 1:**
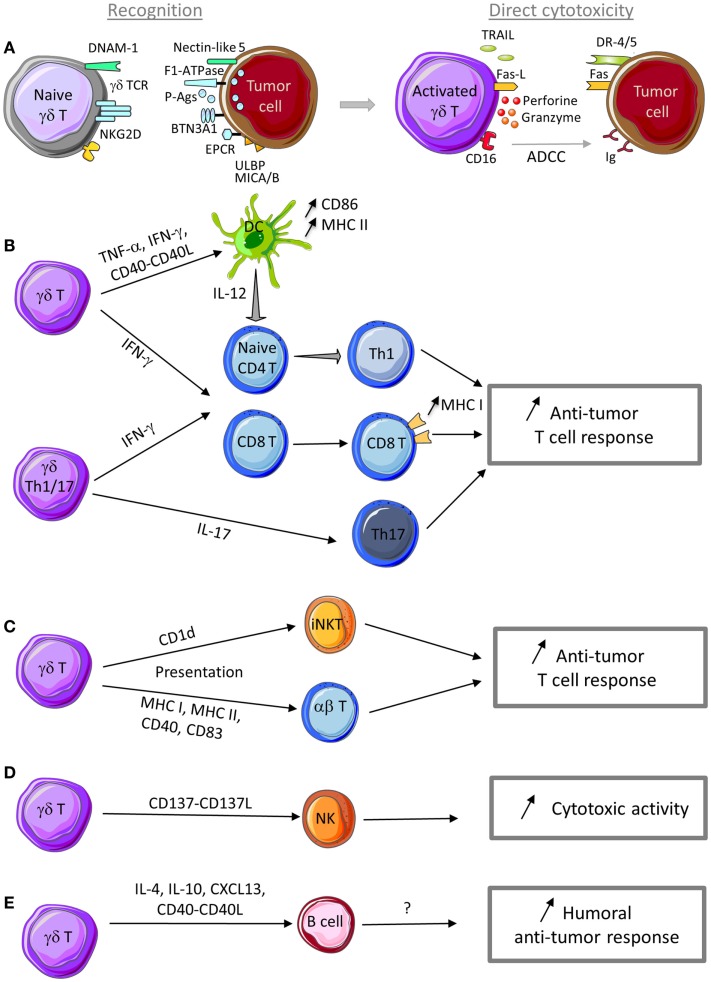
**Anti-tumor functions of γδ T cells**. **(A)** γδ T cells can recognize tumor cells through interaction with (i) TCR ligands, such as phosphoantigens (P-Ags), F1-ATPase, BTN3A1, EPCR, …, and (ii) innate receptor ligands, such as ULBP, MICA/B, and nectin-like 5. Following sensing of tumor antigens or stress signals, γδ T cells are activated and can kill tumor cells through cytotoxic mechanisms that rely on the perforin/granzyme pathway, the death receptor pathway in response to TRAIL or Fas-L expression, and ADCC in the presence of tumor-specific antibodies. **(B)** γδ T cell activation leads to TNF-α and IFN-γ production and CD40-L expression that promote DC maturation and T cell differentiation into Th1 cells. IL-17-producing γδ Th17 cells favor Th17 effector cell development. Th1 and Th17 effector T cells display anti-tumor functions to control tumor development. **(C)** Through a trogocytosis mechanism, activated γδ T cells can capture and express CD1d molecules and then promote iNKT cell activation. Activated γδ T cells can also display APC functions (MHC I and II, CD40, CD83, and CD86 expression) and activate both naive and effector T cells with cytotoxic activity against tumor cells. **(D)** Activated γδ T cells can provide a co-stimulatory signal to NK cells through CD137L expression to promote their anti-tumor activity. **(E)** In the presence of specific signals, activated γδ T cells can display a Tfh profile (i.e., IL-4, IL-10, and CXCL13 production and CD40-L expression) to help B cell antibody production. Although not yet demonstrated, production of antibodies against specific tumor antigens could be involved in the humoral anti-tumor response.

Interestingly, Thedrez et al. demonstrated that expansion of phosphoantigen-activated Vγ9Vδ2 T cells from peripheral blood mononuclear cells (PBMCs) in the presence of IL-21 and IL-2 promotes their cytolytic function (Th1 function), with increased expression of CD56 and several lytic molecules and also higher tumor-induced degranulation capacity (68). However, IL-21 can also promote differentiation of Vγ9Vδ2 T cells toward a Tfh-like phenotype. Indeed, activation of purified Vγ9Vδ2 T cells with phosphoantigens in the presence of IL-21 induces Tfh-associated features, as indicated by the expression of the BCL-6 transcription factor, ICOS, CD40-L, and CXCR5 as well as IL-21R, CD244, CXCL10, and CXCL13 and their trafficking to lymph node germinal centers (59). Both soluble and contact-dependent mechanisms seem to be involved in the B cell helper activity of Tfh-like Vγ9Vδ2 T cells. Indeed, Ig production is consistently impaired by inhibition of CD40-L and ICOS interaction with their respective receptor and ligand or by neutralization of IL-4 and IL-10 (58). It would be interesting to determine whether the interaction between Tfh-like Vγ9Vδ2 T cells and B cells in reactive tumor-associated lymphoid tissues might positively affect the production of high affinity antibodies against tumor antigens, thus favoring antibody-dependent cell cytotoxicity (ADCC) mechanisms (Figure 1E).

Besides these effects on the cytotoxic activity and B cell helper functions of Vγ9Vδ2 T cells, our preliminary data suggest that IL-21 might also confer some regulatory functions to γδ T cells. Overall these data suggest that IL-21 together with environmental signals can strongly influence Vγ9Vδ2 T cell functions by polarizing them toward Th1-, Tfh-, or Th1/Treg-like T cells.

Other co-signals can induce the polarization of Vγ9Vδ2 T cells into Treg cells. Particularly, when they are activated by IPP in the presence of IL-15 and TGF-β, Vγ9Vδ2 T cells express the FOXP3 transcription factor and display regulatory/immunosuppressive activity as demonstrated by their capacity to suppress the proliferation of anti-CD3/anti-CD28-stimulated PBMCs (60). However, they do not simultaneously display regulatory and Th1-like effector functions, differently from regulatory γδ T cells developed in the presence of IL-21. Interestingly, treatment with decitabine (a DNA hypomethylating agent) and IL-15/IL-2/transforming growth factor-β (TGF-β) associated with phosphoantigen activation facilitates the induction of the immunosuppressive functions of Vγ9Vδ2 T cells derived from human PBMCs and favors the regulatory activity of Vγ9Vδ2 T cells (69).

First established for murine γδ T cells (67), the production of IL-17 by human γδ T cells was also recently demonstrated (70). In both mouse and human, IL-7 promotes substantially an expansion of IL-17-producing γδ T cells (71). Moreover, several studies have shown that when cultured in the presence of various combinations of cytokines, naive Vγ9Vδ2 T cells acquire an IL-17-secreting Th17-like phenotype or a mixed Th1/Th17 phenotype and produce both IFN-γ and IL-17 (61–63). Human cord blood-derived Vγ9Vδ2 T cells stimulated with HMBPP require IL-6, IL-1β, and TGF-β to differentiate into γδTh17 cells, whereas, differentiation into γδTh1/Th17 cells needs also IL-23 (62, 63). In adults, differentiation of naive γδ T cells into memory γδTh1/Th17 T cells and γδTh17 T cells requires IL-23, IL-1β, and TGF-β, but not IL-6. γδTh17 cells can also produce IL-22 (especially cells in the cord blood) (62, 63). Recently, Wu et al. demonstrated that, in a colorectal cancer model, activated inflammatory DCs polarize Vγ9Vδ2 cells into γδTh17 cells that secrete high amount of IL-17, but also IL-8, TNF-α, and granulocyte macrophage colony-stimulating factor (GM-CSF) in an IL-23- dependent manner (64).

Besides their T cell effector functions, phosphoantigen-activated Vγ9Vδ2 T cells can express lymph node migration receptors (e.g., CXCR5) and display several hallmarks of professional APCs, such as up-regulation of MHC class I and II molecules and of the co-stimulatory molecules CD40 and CD83 and also the ability to phagocytose and process antigens and to activate naive αβ T cells (72–74) (Figure 1E). These observations are based on results obtained *in vitro*. The APC functions of γδ T cells *in vivo* have not been evaluated and remain to be demonstrated.

Moreover, similarly to αβ T cells, the differential induction of specific effector functions may also depend on the innate immunity receptor class that is engaged and the nature of the cytokine stimuli. For example, NKG2D engagement triggers the induction of human Vγ9Vδ2 T cell cytotoxic functions, thereby influencing the fate of target cells (lysis or survival), but has limited effects on cytokine production (21). Similarly, type I IFN-released by stimulated myeloid and plasmacytoid DCs induces exclusively IFN-γ, but no TNF-α, production by human Vγ9Vδ2 T cells (65).

In conclusion, Vγ9Vδ2 T cells display a surprisingly broad array of functional activities. One essential question is to determine whether such functional plasticity is an intrinsic feature of the whole Vγ9Vδ2 T cell population or whether it is restricted to specific Vγ9Vδ2 T cell subsets. This is an important issue, because it could directly affect γδ T cell-based therapeutic strategies. Indeed, boosting γδ T cell regulatory activity is suitable in some instances (i.e., autoimmune disease), conversely optimizing, for example, their APC or cytotoxic functions could be more important for the treatment of tumors or infections. In terms of cytokine production and cytotoxic activity, Vγ9Vδ2 T cells can be divided in different subsets based on the expression of cell surface markers. Upon *in vitro* activation and extended culture in the presence of IL-2, naive Vγ9Vδ2 T cells (CD27+CD45RA+) can sequentially differentiate into TCM (CD27+CD45RA−), TEM (CD27−CD45RA−), and TEMRA (CD27−CD45RA+) cells. CD45RA−CD27− TEM cells show the highest IFN-γ secretion, while CD45RA+CD27− TEMRA cells are characterized by a strong cytotoxic activity. In contrast, naive CD45RA+CD27+ Vγ9Vδ2 T cells display very low, if any, functional activity (75). Studies using cell sorter-purified Vγ9Vδ2 T cell subsets have determined that only naive CD45RA+CD27+ Vγ9Vδ2 T cells can differentiate into IL-17-producing cells when exposed to IL-1β, IL-6, IL-23, and TGF-β (61). IL-17-producing Vγ9Vδ2 T cells display a TEMRA phenotype, promote neutrophil migration through production of CXCL8 and up-regulate β-defensin production in epithelial cells (61). Similarly, Vγ9Vδ2 T cell cytotoxic activity can be assigned to specific subsets, especially to (CD45RA+CD27−) TEMRA and (CD56+CD16+) cells (75–77), but their clonal plasticity remains uncertain.

In addition, whether a given Vγ9Vδ2 T cell phenotype induced by specific environmental stimuli, such as cytokines, is stable or reversible, remains to be investigated. Although the expression of lineage-associated transcription factors in Vγ9Vδ2 T cells has been assessed in some studies, so far no clear correlation between the expression of transcription factors and a specific stable cytokine profile has been reported.

Finally, most of these studies concerned the Vγ9Vδ2 T cell subset thus raising the question of whether other human or mouse γδ T cell populations display similar plasticity. Ribot and collaborators have reported that also human Vγ1 and Vγ2 thymocytes show functional phenotypic plasticity and can differentiate into cytotoxic type 1 effector cells following IL-2 or IL-15 stimulation (66) but no investigation was reported on other human γδ T cell subsets.

### Plasticity of mouse γδ T cells

In mice, several studies demonstrated that γδ thymocytes are functionally pre-committed and polarized in term of cytokine production (78–80). During fetal development, γδ T cells are generated from two waves of thymocytes that express invariant TCR. The first group migrates into the skin (Vγ5Vδ1 DETC) and is programed to produce IFN-γ; the second group migrates into the vaginal epithelium and the peritoneal cavity (Vγ6Vδ1 subset) and is programed to produce IL-17 (33, 81). Other γδ T cell subsets appear postnatally in the thymus and express TCR with various Vδ and Vγ combinations. In adult mice, these cells are found in all lymphoid organs and below the epithelium or mucosal surfaces of many tissues, including the small intestine and lung. Most of them display a programed polarization acquired during thymic selection (33, 81) through a process regulated by TCR (78–80) and co-receptor signaling (81). Thus, γδ T cell differentiation into IFN-γ-producing cells require TCR and CD27 signals (78–80). CD27, a member of the tumor necrosis factor receptor family, regulates the balance between IFN-γ and IL-17 producing γδ T cell subsets (82). CD27+ γδ T cells are committed to express IFN-γ genes, whereas, CD27− γδ T cells display a permissive chromatin configuration at loci encoding IFN-γ and IL-17 as well as their regulatory transcription factors. They can thus differentiate into both IFN-γ- and IL-17-producing cells (82). It has also been shown that IL-23 in combination with IL-1β promotes IL-17, IL-21, and IL-22 expression by mouse γδ T cells in the absence of additional signals; however, the authors did not investigate CD27 expression in this setting (67).

Altogether, these results suggest that mouse γδ T cells have a low plasticity compared to human γδ T cells. Nevertheless further investigation on mouse and human γδ T cell functional plasticity are required to better characterize the molecular mechanisms and the precise role of each γδ T cell subset in the immune response and in pathologic conditions in order to improve γδ T cell-based therapies.

## Impact of γδ T Cells on the Tumor Immune Response

γδ T cells can: (i) detect and sense any type of stress through a MHC-independent mechanism, (ii) produce huge quantities of pro-inflammatory cytokines, and (iii) exert potent cytotoxic activity against a broad panel of tumors. For these reasons, γδ T lymphocytes are key players in the tumor immune response. Like other cytotoxic effectors, γδ T cells directly participate in the elimination of tumor cells, but they also control indirectly the tumor immune response by modulating the activity and functions of other immune cells. In this section, we will summarize both pro- and anti-tumor activities of γδ T cells by focusing mainly on their tumor recognition mechanisms and the triggered biological responses.

### Anti-tumor activity of γδ T cells

#### Mechanisms of tumor cell recognition

Similarly to any other T cell population, γδ T cell activation and acquisition of effector functions are triggered by TCR engagement (Figure 1A). Specifically, γδ TCR recognize molecules that are over-expressed in stress conditions. In normal cells, the concentration of metabolites of the isoprenoid pathway, such as IPP, is too low to be sensed as a danger signal by Vγ9Vδ2 T cells. Deregulation of the isoprenoid pathway in some tumors leads to IPP over-production that is detected and considered as a tumor antigen by Vγ9Vδ2 TCR (40, 83). Similarly, incubation of tumor cells with bisphosphonates that inhibit the farnesyl pyrophosphate synthase enzyme in the isoprenoid pathway leads to IPP accumulation and makes tumor cells more sensitive to Vγ9Vδ2 T cell cytotoxicity (84–86). Several reports have shown that phosphoantigens need to interact with specific proteins to be recognized by TCR and to activate Vγ9Vδ2 T cells. First, Mookerjee-Basu et al. showed that F1-ATPase, which is expressed on the surface of some tumor cells, binds to the adenylated derivative of IPP and is involved in triggering Vγ9Vδ2 T cell activation and anti-tumoral activity (87, 88). More recently, it was reported that butyrophilin 3 A1 (BTN3A1) can contribute to γδ T cell activation by sensing changes in phosphoantigen concentration within tumor cells. Specifically, phosphoantigen binding to the intracellular domain of BTN3A1 could initiate a cascade of events that result in extracellular changes or cell surface rearrangements (including immobilization of BTN3A extracellular domain) and lead to Vγ9Vδ2 T cell activation (89, 90). Dechanet-Merville and collaborators found that a human δ2 negative T cell subset recognizes both CMV-infected and transformed cells through the interaction between the endothelial protein C receptor (EPCR) and the TCR (91). EPCR is over-expressed in CMV-infected endothelial cells and transformed cells and it is conceivable that it might act as a determinant of stress surveillance during epithelial cell transformation to communicate a state of “dysregulated self” to γδ T cells.

In addition to TCR engagement, stimulation of NKR expressed by γδ T cells and particularly engagement of NKG2D receptor can also efficiently trigger the anti-tumor functions of γδ T cells. NKG2D is expressed by Vγ9Vδ2 T cells and binds to non-classical MHC molecules of the MIC and ULBP families that are expressed by tumor cells (18, 20, 21). Ligand binding to NKG2D induces the release of IFN-γ and TNF-α, increases the expression of CD25, the α chain of the IL-2 receptor and promotes γδ T cell cytolytic activity (21). In particular, ULBP molecules have been involved in the recognition by Vγ9Vδ2 T cells of leukemia and lymphoma (92) and also of solid tumors, such as ovarian and colon carcinomas (93, 94). For instance, ULBP1 expression level determines lymphoma susceptibility to γδ T cell-mediated cytolysis upon NKG2D binding (92). ULBP4 also can bind to Vγ9Vδ2 TCR and thus induce the cytotoxic activity of Vγ9Vδ2T cells toward tumor cells through both TCR and NKG2D engagement (22). More recently, Lamb and collaborators have shown that temozolomide (TMZ), the main chemotherapeutic agent used to treat glioblastoma multiforme (GMB), increases the expression of stress-associated NKG2D ligands on TMZ-resistant glioma cells, potentially making them more susceptible to γδ T cell recognition and lysis (95). Furthermore, as described for Vγ9Vδ2 T cells, recognition of MICA, MICB, or ULBP expressed on cancer cells by human Vγ1δ1 T lymphocytes can trigger or increase their cytolytic activity against tumor cells that express NKG2D ligands (23, 96). Indeed, ULBP and MICA interact with NKG2D or TCR on Vδ1 γδ T cells and induce their activation. However, MICA binds in mutually exclusive manner to NKG2D and TCR, suggesting that the two receptors might be sequentially engaged following recognition of target tumor cells (97).

DNAM-1 (also called CD226) is another NKR involved in the regulation of the cytotoxic activity of γδ T cells. It is expressed on the surface of both Vγ9Vδ2 and γ1 T cell populations and its ligand nectin-like-5 has been detected on certain tumors. DNAM-1 cooperates with TCR and NKG2D signaling in γδ T cells to positively regulate their IFN-γ production and cytotoxic activity against tumor cells (98, 99).

Like NK cells, human γδ T cells also express the CD16 (FcγRIII) receptor that binds to the Fc portion of immunoglobulin G (IgG). CD16 expression on Vγ9Vδ2 T cells can be up-regulated following stimulation with phosphoantigens (100). Its engagement leads to ADCC (101), a process that can result in lysis of tumor cells bound by specific antibodies. Indeed, several *in vitro* studies have clearly shown that γδ T cells are activated through CD16 and mediate ADCC of tumor cells in the presence of therapeutic anti-tumor monoclonal antibodies, such as rituximab, trastuzumab, atumumab, and alemtuzumab (102–105). Reinforcing the relevance of such *in vitro* data, it has been shown that stimulated γδ T cells increase the efficacy of Trastuzumab in Her2+ breast cancer patients (105).

#### Impact on immune cell activity

In addition to these direct effects against tumor cells, γδ T cells can also control indirectly the anti-tumor immune response by promoting the recruitment and modulating the activation of other cell types in the tumor microenvironment, such as DCs, NK cells, and effector T cells (Figures 1B–D).

In the presence of tumor cells, or following stimulation with TCR agonists, NKG2D ligands, cytokines (such as IL-12 and IL-18), or DNAM-1 engagement, human γδ T cells produce IFN-γ and TNF-α (21, 56, 94, 106–108). These two cytokines can inhibit tumor growth through several mechanisms, but especially by enhancing CD8 T cell anti-tumor activity (Figure 1B) and by inhibiting tumor angiogenesis (109–111). Mouse γδ T cells also are an important and early source of IFN-γ within the tumor microenvironment where IFN-γ enhances MHC class I expression on tumor cells and CD8+ T cell responses (112–114). Altogether these findings suggest that both human and mouse γδ T cells positively influence the anti-tumor immune response by increasing the adaptive anti-tumor immunity (115) (Figure 1B).

As previously mentioned, both mouse and human γδ T cells could be an important source of IL-17. This cytokine plays an essential role in the host defense against microbial infections, but also in autoimmune disorders and cancer (116). IL-17 contribution to the tumor immune surveillance is still controversial. Indeed, IL-17 has often been described as a cytokine with pro-tumor properties, but several studies highlighted that it can also display anti-tumor functions (117). Therefore, IL-17 heterogeneous sources and, perhaps, targets in the tumor microenvironment may determine whether it will negatively or positively affect tumor growth. In human, the majority of αβ and γδ Th17 cell populations that produce IL-17 also concomitantly produce IFN-γ (63) and the anti-tumor functions of IL-17-producing αβ T cells strongly depend on IFN-γ (118). Moreover, IL-17-producing αβ T cells stimulate the release of several cytokines (such as IL-6, IL-12, CXCL9, and CXCL10) by immune or cancer cells, leading to DC maturation or effector T cell recruitment to the tumor, and as a consequence, to an increase of the anti-tumor immunity (119, 120) (Figure 1B). It is likely that γδ Th17 cells might do the same, but this remains to be formally demonstrated.

Importantly, in mice, IL-17-producing γδ T cells (Vγ4+ and Vγ6+) contribute to chemotherapy efficacy because they are required for the priming of IFN-γ-secreting tumor-specific T cells. In this context, γδ T cells are considered as part of the innate immune response that is involved in the subsequent specific anti-tumor T cell response following treatment with chemotherapeutic agents (121, 122). Nevertheless, it is not known whether human IL-17 γδ T cells also contribute to the efficacy of anti-cancer chemotherapy and whether combination treatments with γδ T cell agonists and anthracyclines could improve the patient outcome.

Dendritic cells are potent inducers of γδ T cell effector functions through their ability to express γδ TCR ligands and to provide co-stimulation signals (123, 124). Inversely, interactions between activated γδ T cells and DCs were shown to induce DC activation and maturation, thus facilitating the establishment of the T cell response (125, 126). Indeed, activated human Vγ9Vδ2 T cells enhance IL-12 production by monocyte-derived DCs through an IFN-γ- and IL-12-mediated positive feedback loop that can then promote naive αβ T cell activation and differentiation into Th1-type cells (127), an effect that may positively influenced the anti-tumor immunity (Figure 1B).

As already mentioned, when activated by phosphoantigens, Vγ9Vδ2 T cells can display APC features and acquire the ability to activate naive and effector T cells (72, 73) (Figure 1C). Similarly, Vγ9Vδ2 T cells can also present antigens to invariant NKT cells (iNKT). Schneiders et al. demonstrated that, when co-cultured with CD1d-positive cells, activated Vγ9Vδ2 T cells uptake CD1d on their membrane through trogocytosis and acquire the capacity to present glycolipid antigens to iNKT cells and activate them (128) (Figure 1C). iNKT cell activation triggers the production of large amounts of cytokines that play an important role in initiating and orchestrating anti-tumor immune responses, such as Th1-biased pro-inflammatory responses.

Natural killer cells also have a role in anti-tumor responses and their activity can be regulated by γδ T cells. When co-localized within tumors, human γδ T cells can provide co-stimulatory signals to NK cells and induce NK cell-mediated killing of tumor cells (129). Indeed, CD137L is expressed on activated γδ T cells and interacts with the cognate receptor CD137 on NK cells, leading to the up-regulation of the activation markers CD25, CD54, CD69, and NKG2D on the surface of NK cells and to the increase of their cytotoxic function, particularly against solid tumors that are usually resistant to NK cytolysis (129) (Figure 1D).

### Pro-tumor activity of γδ T cells

**Figure 2:**
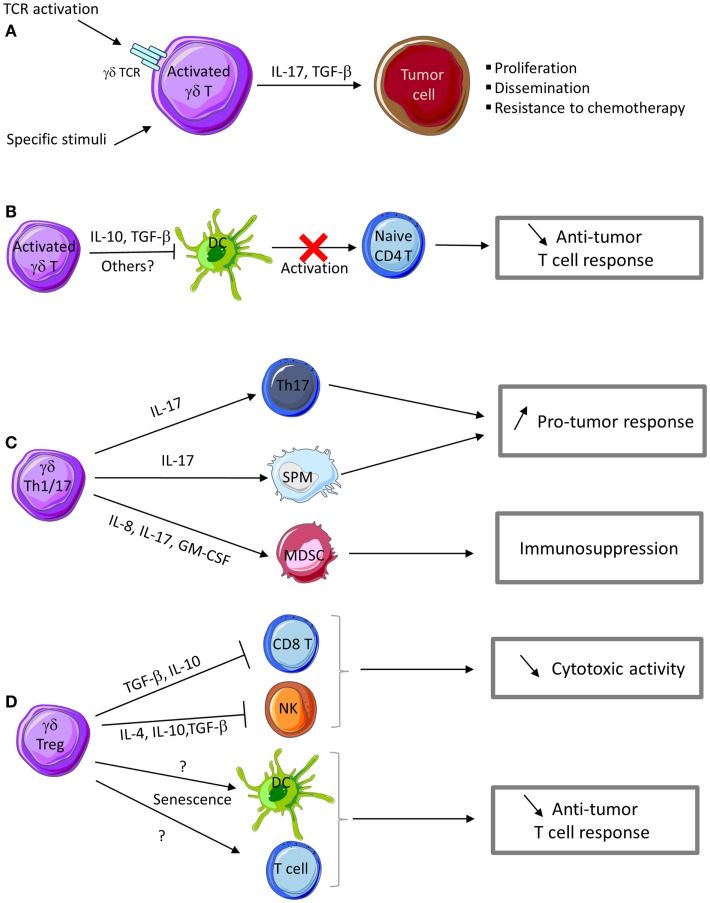
**Pro-tumor functions of γδ T cells**. **(A)** Activation of γδ T cells in the presence of specific stimuli can promote their polarization into Th17- or Treg-like cells that produce IL-17 and TGF-β, thus favoring tumor cell proliferation and dissemination. IL-17 produced by γδ T cells confers chemotherapy resistance to tumor cells. **(B)** Activated γδ T cells can inhibit DC maturation and their APC functions, thus impairing naive T cell activation and differentiation into effector T cells. **(C)** IL-17 produced by γδ Th17 cells promotes the development of Th17 cells with pro-tumor functions. γδ Th17 cells also produce a cocktail of cytokines and chemokines involved in the recruitment of myeloid-derived suppressive cells (MDSC) and small peritoneal macrophages (SPM) with immunosuppressive and pro-tumor functions. **(D)** γδ Treg cells produce cytokines (IL-4, IL-10, and TGF-β) and other immunosuppressive factors that impair CD8 T and NK cell cytotoxic activity. γδ Treg cells can also promote senescence of DC or αβ T cells and consequently favor tumor growth.

In some conditions, γδ T cells can also promote tumor growth via regulatory functions that impair the anti-tumor immune responses (Figure 2).

#### Human Vγ9V δ2 T cells

Vγ9Vδ2 T cells with immunosuppressives functions may play an important role in human cancers. Upon activation, human peripheral Vγ9Vδ2 T cells also can express IL-4, IL-10, and TGF-β and inhibit T cell proliferation, thus developing a regulatory profile that may play a role in the suppression of anti-tumor responses (130). Indeed, depending on the context, Vγ9Vδ2 T cells may display a Th1-, Th2-, Th17-, or Th1/reg-like profile and synthetize IFN-γ, IL-4, IL-17 or IL-10, and TGF-β, respectively.

While IL-4 is a cytokine involved in Th2 responses (which are not appropriate for anti-tumor immunity), IL-10 and TGF-β are cytokines with immunosuppressive functions and thus could be involved in the pro-tumor activities of γδ T cells. TGF-β has a crucial role in tumor development because it can promote tumor cell invasiveness and metastasis formation mainly by modulating the immune system and the tumor microenvironment (Figure 2A). The most important mechanisms of tumor progression linked to TGF-β activities are the epithelial-to-mesenchymal transition (EMT), immune system evasion, and promotion of cancer cell proliferation by modulation of the tumor microenvironment (131). The expression of IL-10 and TGF-β is frequently increased in various cancer types. IL-10 directly affects APC function by inhibiting the expression of MHC and co-stimulatory molecules, which induces immune suppression or tolerance (Figure 2B). Additionally, IL-10 down-regulates the expression of Th1 cytokines and induces T-regulatory responses.

IL-17 plays a dual role by promoting both tumor growth and anti-tumor immunity, depending on the tumor type, stage, and target cells present in tumor microenvironment. The number of IL-17-producing cells is increased in cancer and this is associated with poor prognosis (117, 132, 133). Several IL-17 activities contribute to tumor progression. In breast cancer, IL-17 can directly promote tumor cell proliferation and dissemination (119) and favor the development of cancer resistance to conventional chemotherapeutic agents, such as docetaxel (133) (Figure 2A). IL-17 can also act on cells in the tumor microenvironment. For instance, IL-17 up-regulates the secretion of pro-angiogenic and pro-tumor factors (e.g., VEGF, IL-6, and IL-8) by stromal cells and fibroblasts, thus promoting angiogenesis and sustained chronic inflammation (119, 120). In colorectal cancer, Vγ9Vδ2 T cells can differentiate into Th17 cells that secrete IL-17 and also IL-8, TNF-α, and GM-CSF and thus contribute to the accumulation of immunosuppressive polymorphonuclear-myeloid-derived suppressor cells (PMN-MDSCs) within the tumor microenvironment and influence the anti-tumor immune response (64) (Figure 2C).

#### Human V δ1 T cells

Besides Vγ9Vδ2 T cells, other human γδ T cell subsets can display immunosuppressive functions. First, Peng et al. demonstrated that Vδ1 γδT cells infiltrating human breast cancer suppress DC maturation and T cell effector functions both *in vitro* and *in vivo*. When stimulated by tumor cells and an anti-CD3 antibody, Vγ1 T cells express IFN-γ and GM-CSF, but not IL-1β, TNF-α, IL-12, IL-2, IL-4, IL-10, or TGF-β (134). Thus neither IL-10 nor TGF-β seems to play a role in this immunosuppressive activity. Although, the involved factor(s) remain to be identified, these authors found that the suppressive activity was in the soluble fraction with a molecular mass higher than 100 kDa and could be inactivated by heat, but not by DNAse or RNAse treatments (134) (Figure 2D). These Vδ1 γδ T cells represent a large percentage of tumor-infiltrating lymphocytes in breast and also in prostate cancer, suggesting that they may play an important role in promoting an immunosuppressive tumor microenvironment. Interestingly, stimulation of suppressive Vδ1 γδ T cells in breast cancer by using a TLR8 agonist reversed the anti-tumor response inhibition (134). More recently, the same group demonstrated that regulatory γδ T cells can induce both T cell and DC senescence. Specifically, regulatory γδ T cells induce senescence of both naive and effector T cells, as indicated by the impaired expression of the co-stimulatory molecules CD27 and CD28 and the low proliferative capacities of both Th1 and Th17 T cell subsets. Senescent T cells and DCs become suppressive cells, further amplifying the immunosuppression mediated by γδ Treg cells (135). Furthermore, Ma and collaborators found that high γδ T cell level in breast cancer tissues is correlated with poor survival and high risk of relapse (136). Similarly, in colon adenocarcinoma, a significant correlation has been observed between presence of γTCR cells and disease stage. These two reports suggest that γδ T cells may have a key prognostic role in colon adenocarcinoma and breast cancers (137).

#### Mouse γδ T cells

γδ T cells with immunosuppressive functions have also been observed in mouse tumor models (138, 139). Seo et al. found that murine γδ T cells that infiltrate tumors arising from B16 melanoma cells produce large amounts of IL-4 and IL-10 and inhibit NK and iNKT cell activity (138) (Figure 2D). They demonstrated that supernatants from these γδ T cells did not affect NK and iNKT cell cytotoxicity, but reduced their proliferation, suggesting that soluble IL-4 and IL-10 could contribute to the inhibition of NK and iNKT cell activity by γδ T cells in this model (138). Additional studies from this group showed that γδ T cells that infiltrate MM2 mammary tumors in mice express IL-10 and TGF-β, but not IFN-γ or IL-4. γδ T cells isolated from these tumors and from the spleen hindered the cytotoxic activity of NK and CD8 T cells. IL-10 and TGF-β neutralization inhibited some of the immunosuppressive effects of these γδ T cells, suggesting the involvement of these cytokines (Figure 2D). Moreover, depletion of IL-10- and TGF-β-secreting γδ T cells by using a specific antibody enhanced the anti-tumor immunity and reduced tumor growth in xenografted mice (139). More recently, Hao et al. using the B16 melanoma model, showed that mouse Vγ1 T cells suppress the anti-tumor functions of the Vγ4 T cell subset, thus promoting tumor growth. Specifically, Vγ1 γδ T cells reduced IFN-γ, perforin, and NKG2D expression in Vγ4 γδ T cells through contact-independent mechanisms involving IL-4 (140). Collectively, these data strongly suggest that within the tumor microenvironment, some mouse γδ T cell populations express IL-4, IL-10, and TGF-β and inhibit the anti-tumor immune response. IL-17-secreting γδ T cells show pro-tumor activity also in mouse models. Recently, Rei et al. demonstrated that murine CD27-Vγ6 T cells that produce IL-17 promote ovarian cancer growth *via* mobilization of small peritoneal macrophages (141) (Figure 2C).

Overall, these findings support the idea that γδ T cells, at least in some cancers, can behave as Tregs or Th17 T cells that impair the anti-tumor immune response and promote tumor growth, through the secretion of different cytokines with regulatory functions or the recruitment of immunosuppressive cells within the tumor microenvironment.

## Conclusion

During the last decade, our knowledge on the role of γδ T cells in the tumor microenvironment has hugely improved. Plasticity of γδ T cells increases the range of their biological responses as different γδ T cell sub-populations can regulate different aspects of the tumor immunity. Functional plasticity also can explain the heterogeneous responses and contradictory functions of this unconventional T cell population in the context of cancer immune surveillance. As discussed in this review, due to the TCR-mediated recognition and activation mechanisms and the fine regulation of their activation through innate and cytokine receptors, γδ T lymphocytes are attractive targets for immunotherapeutic protocols with the final objective of boosting the anti-tumor immune response. Several clinical trials have already assessed γδ T cell-based immunotherapy in patients with advanced hematological malignancies and solid cancers with encouraging results. However, high density of γδ T cells in the breast and colon tumor microenvironment has been associated with poor clinical outcome. We are convinced that a better characterization of the mechanisms regulating their polarization should allow the development of optimal therapeutic strategies to favor the expansion of γδ T cell populations with anti-tumor rather than pro-tumor functions.

## Conflict of Interest Statement

The authors declare that the research was conducted in the absence of any commercial or financial relationships that could be construed as a potential conflict of interest.
